# Development of an enzyme-linked immunosorbent assay based on viral antigen capture by anti-spike glycoprotein monoclonal antibody for detecting immunoglobulin A antibodies against porcine epidemic diarrhea virus in milk

**DOI:** 10.1186/s12917-023-03605-4

**Published:** 2023-02-11

**Authors:** Rui Li, Ying Wen, Lei Yang, Qi-sheng Qian, Xin-xin Chen, Jia-qing Zhang, Xuewu Li, Bao-song Xing, Songlin Qiao, Gaiping Zhang

**Affiliations:** 1 Key Laboratory of Animal Immunology of the Ministry of Agriculture, Henan Provincial Key Laboratory of Animal Immunology, Henan Academy of Agricultural Sciences, Zhengzhou, 450002 Henan China; 2 Institute of Animal Husbandry and Veterinary Science, Henan Academy of Agricultural Sciences, Zhengzhou, 450002 Henan China

**Keywords:** PEDV, IgA antibody detection, ELISA, S protein, mAb

## Abstract

**Background:**

Porcine epidemic diarrhea (PED), caused by PED virus (PEDV), is a severe enteric disease burdening the global swine industry in recent years. Especially, the mortality of PED in neonatal piglets approaches 100%. Maternal antibodies in milk, particularly immunoglobulin A (IgA) antibodies, are of great importance for protection neonatal suckling piglets against PEDV infection as passive lactogenic immunity. Therefore, appropriate detection methods are required for detecting PEDV IgA antibodies in milk. In the current study, we prepared monoclonal antibodies (mAbs) against PEDV spike (S) glycoprotein. An enzyme-linked immunosorbent assay (ELISA) was subsequently developed based on PEDV antigen capture by a specific anti-S mAb.

**Results:**

The developed ELISA showed high sensitivity (the maximum dilution of milk samples up to 1:1280) and repeatability (coefficient of variation values < 10%) in detecting PEDV IgA antibody positive and negative milk samples. More importantly, the developed ELISA showed a high coincidence rate with a commercial ELISA kit for PEDV IgA antibody detection in clinical milk samples.

**Conclusions:**

The developed ELISA in the current study is applicable for PEDV IgA antibody detection in milk samples, which is beneficial for evaluating vaccination efficacies and neonate immune status against the virus.

**Supplementary Information:**

The online version contains supplementary material available at 10.1186/s12917-023-03605-4.

## Background

Porcine epidemic diarrhea (PED), characterized by vomiting, watery diarrhea, and dehydration, is a highly contagious swine enteric disease [1]. Pigs of all ages suffer from PED, while neonatal piglets are susceptible to PED with up to 100% mortality [2, 3]. PED was first reported in England in 1971 [4], and spread to other European and Asian countries [5]. Since 2010, PED re-emerges and becomes prevalent in China [6, 7]. In 2013, PED broke out in the USA and led to seven million deaths of pigs, corresponding to almost 10% of the national swine population [8, 9]. To date, PED is epidemic in numerous pig-raising countries and causes enormous economic losses to the global swine industry [10].

Vaccination remains as an effective strategy to prevent and control PED [10, 11]. However, due to their immature immune systems, neonatal suckling piglets acquire passive lactogenic immunity against infection by PED causative agent, PED virus (PEDV) [12]. Maternal antibodies in colostrum and milk play a critical role in lactogenic immunity. As immunoglobulin A (IgA) antibodies are dominant and persistent in milk throughout lactation [13, 14], their appropriate measurement is necessary to evaluate vaccination efficacies and neonate immune status against PEDV.

PEDV is an enveloped, single-stranded positive-sense RNA virus belonging to the order *Nidovirales*, family *Coronaviridae*, genus *Alphacoronavirus*. The PEDV genome is approximately 28 kb in length, and encodes four structural proteins, one accessory protein and 16 nonstructural proteins [15]. Among them, PEDV spike (S) glycoprotein is a class I virus fusion protein on virion surface [16], and can be further divided into the S1 subunit involved in receptor recognition and the S2 subunit mediating membrane fusion [17]. In addition, the S protein contains multiple antigenic epitopes, which induce immune responses and neutralizing antibodies [18, 19].

In order to establish a reliable diagnostic tool for PEDV IgA antibodies in milk, we firstly prepared monoclonal antibodies (mAbs) against recombinant PEDV S protein in the current study. Subsequently, we developed an enzyme-linked immunosorbent assay (ELISA) based on PEDV antigen capture by a specific anti-S mAb. The ELISA showed good sensitivity and repeatability in detecting PEDV IgA antibody positive and negative milk samples, and a high coincidence rate with a commercial ELISA kit for detecting clinical milk samples.

## Results

### Preparation of anti-PEDV mAbs

**Fig. 1:**
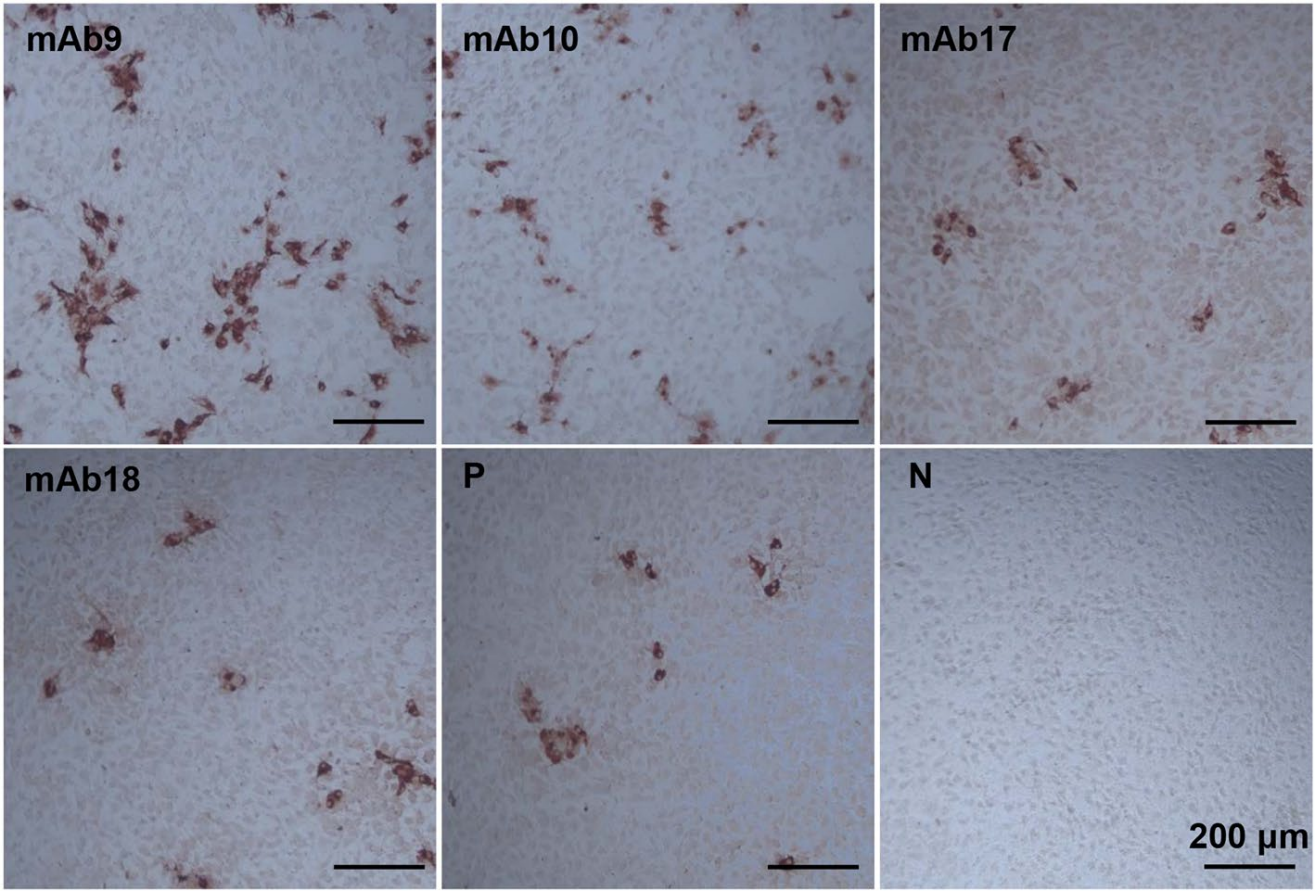
Screen of anti-PEDV mAbs by IPMA. The anti-PEDV mAbs strongly bound to the CPE regions in the PEDV-infected IPEC-J2 cells (brown). Representative images were shown for the mAbs 9, 10, 17 and 18, and PEDV positive (P) and negative (N) sera. Scale bars, 200 μm

To prepare the mAbs specific for PEDV, we immunized mice with PEDV and screened via immunoperoxidase monolayer assay (IPMA). Positive hybridoma cell lines were subcloned, and four mAbs named as mAb9, mAb10, mAb17 and mAb18 were chosen for subsequent experiments. Figure 1 showed specific binding of these four mAbs to the cytopathic effect (CPE) regions in the PEDV-infected porcine intestine epithelial IPEC-J2 cells.

### Identification and characterization of anti-S mAbs

**Fig. 2:**
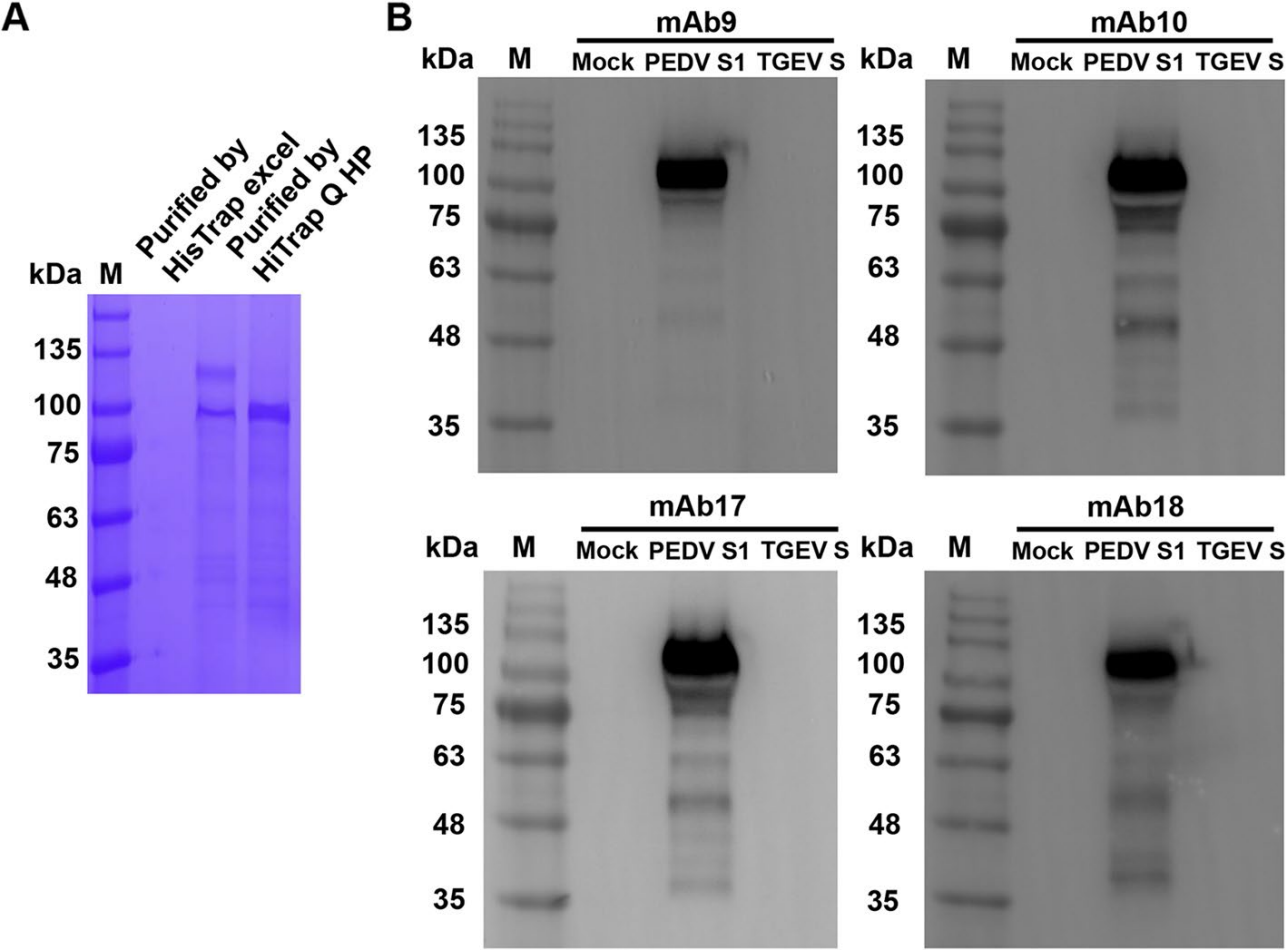
Identification of anti-S mAbs by Western blot. **A** The recombinant PEDV S1 subunit was purified by HisTrap excel prepacked column and HiTrap Q HP prepacked column, and then analyzed by SDS-PAGE. **B** The purified PEDV S1 subunit was applied to SDS-PAGE, transferred to PVDF membranes, and detected by the mAbs 9, 10, 17 and 18. In parallel, mock control and recombinant TGEV S protein were applied. The results were visualized with HRP-conjugated goat anti-mouse IgG and Solarbio ECL plus reagent. The original gel and blots are presented in Additional file 2

Next, we determined whether the chosen anti-PEDV mAbs recognized PEDV S protein. Recombinant PEDV S1 subunit was prepared and obtained with a high purity (~ 110 kDa, Fig. 2A). As shown in Fig. 2B, the purified PEDV S1 subunit was specially bound by all the four mAbs in Western blot, while mock control or recombinant S protein from another Alphacoronavirus transmissible gastroenteritis virus (TGEV) wasn’t, indicating that these mAbs were capable of recognizing PEDV S protein in a specific manner.

**Fig. 3:**
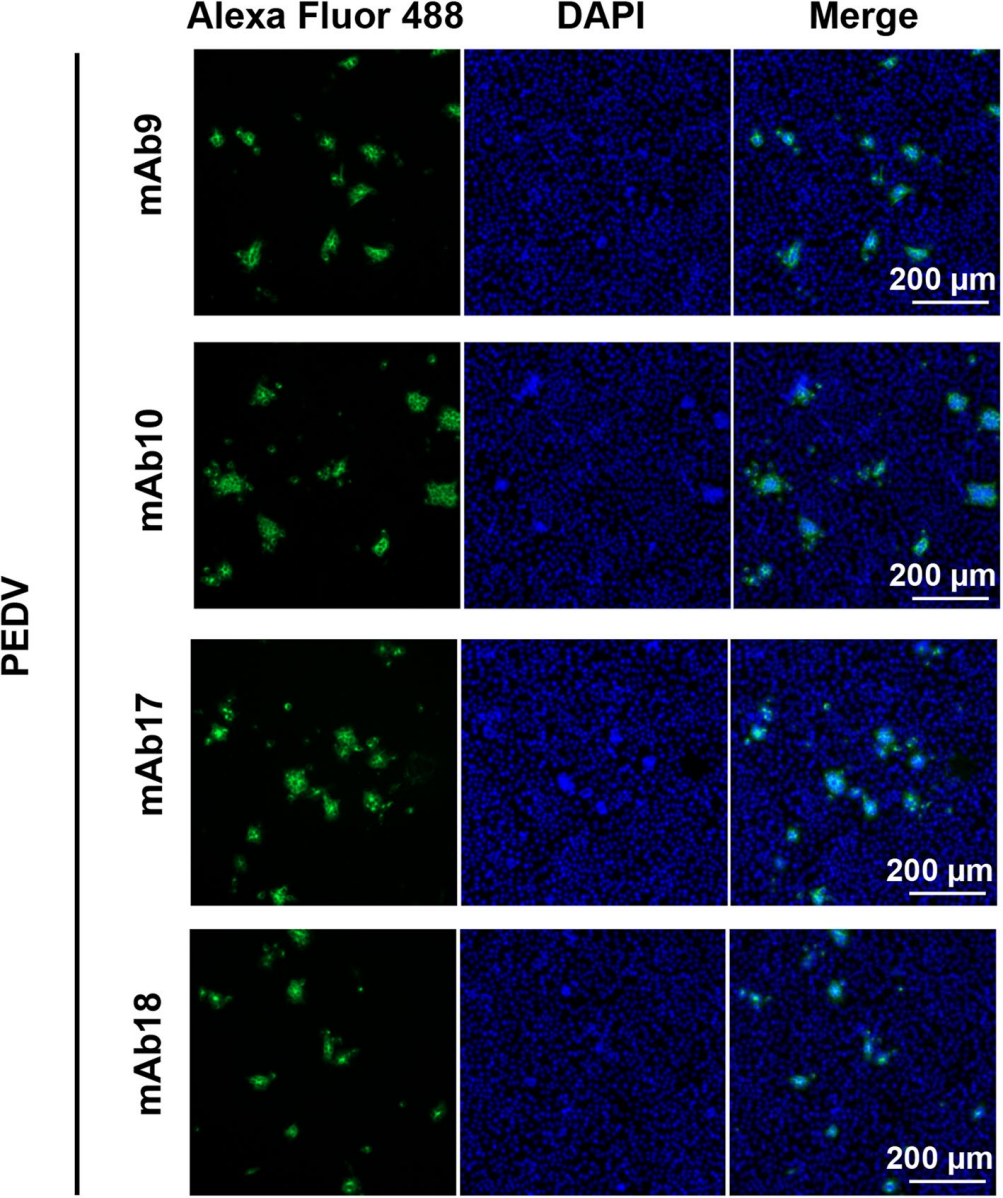
IFA analyses of PEDV-infected Vero cells recognized by the anti-S mAbs. The Vero cells with the 80% confluence were inoculated with PEDV until exhibiting obvious CPEs. After fixed and blocked, the cells were incubated with the anti-S mAbs and Alexa Fluor 488-donkey anti-mouse IgG. The cell nuclei were stained using DAPI and the cells were visualized by Leica fluorescence microscopy. Representative images were shown. Scale bars, 200 μm

To further demonstrate their recognition capabilities, the PEDV-infected African green monkey kidney epithelial Vero cells were monitored by indirect immunofluorescence assay (IFA) using the anti-S mAbs. Figure 3 showed that the anti-S mAbs strongly bound to the CPE regions in the PEDV-infected Vero cells whereas not to the uninfected ones. These results revealed that the mAbs specially recognized PEDV S protein and virions during infection.

After characterization, the subtype of mAb9 heavy chain was identified as IgG2b, that of mAb10 and mAb18 was IgG2a, and that of mAb17 was IgG1 for further use.

### Selection of the mAb9 for establishing ELISA

**Fig. 4:**
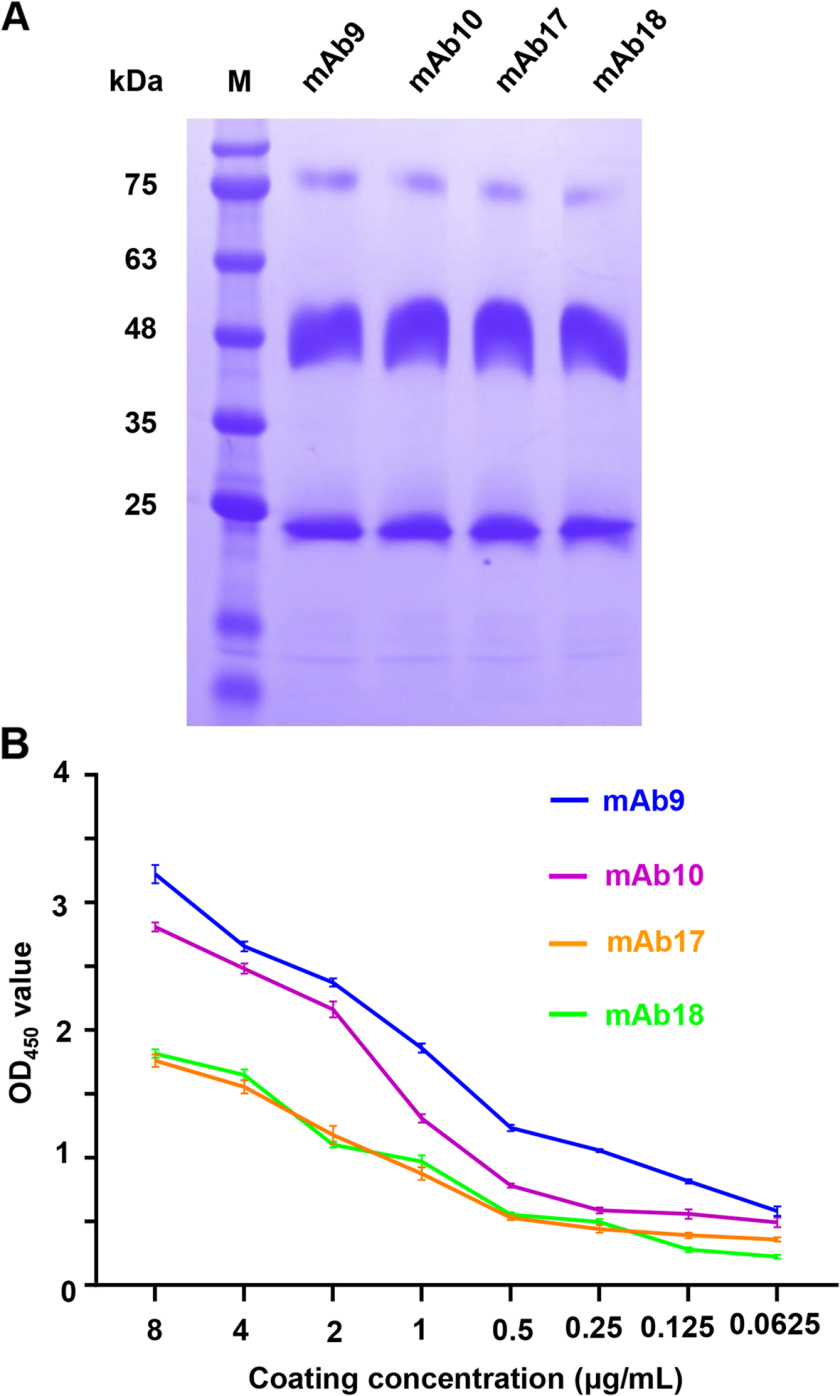
Selection of the mAb9 for ELISA development. **A** The purified anti-S mAbs were analyzed by SDS-PAGE. The original gel is presented in Additional file 2. **B** Selection of the optimal anti-S mAb for developing ELISA. Each purified anti-S mAb was two-fold serially diluted as 8, 4, 2, 1, 0.5, 0.25, 0.125 and 0.0625 μg/mL, and coated on 96-well ELISA plates. Then, the plates were blocked and incubated with inactivated cell-cultivated PEDV supernatants (1 mg/mL). The plates were incubated with the rabbit anti-PEDV pAbs and HRP-conjugated goat anti-rabbit IgG, and reacted with TMB substrate solution. After stopped, OD_450_ values were measured on the BMG-Labtech microplate reader. The error bars indicate SD values

The anti-S mAbs were purified from ascites, and Fig. 4A showed that heavy (~ 50 kDa) and light chains (~ 25 kDa) were primarily visualized for all the four mAbs on sodium dodecyl sulfate–polyacrylamide gel electrophoresis (SDS-PAGE), indicating their high purities.

To select the optimal one for establishing ELISA, each purified anti-S mAb was diluted, coated and tested for PEDV antigen capture. PEDV antigen capture by the mAb9 was shown with the highest value of optical density at 450 nm (OD_450_) at each coating concentration (Fig. 4B and Table S1) and therefore chosen to establish ELISA.

### Development of ELISA

ELISA was established based on PEDV antigen capture by the mAb9 and optimized by checkerboard titration. As shown in Table S2, the optimal concentrations of the mAb9 for coating and inactivated cell-cultivated PEDV supernatants for viral antigen capture are 0.5 μg/mL and 0.5 mg/mL, respectively. The optimal coating condition of the mAb9 was at 4 °C for 12 h (Table S3). The optimal blocking solution was phosphate buffered saline (PBS) containing 5% bovine serum albumin (BSA), and the optimal blocking condition was at 37 °C for 1.5 h (Table S4). The optimal dilution and incubation time of milk samples were 1:40 and at 37 °C for 30 min (Tables S5 and S6). The optimal dilution and incubation time of horseradish peroxidase (HRP)-conjugated goat anti-pig IgA were 1:20,000 and at 37 °C for 30 min (Tables S7 and S8). The optimal reaction condition of 3, 3’, 5, 5’-tetramethylbenzidine (TMB) substrate solution was in dark at room temperature (RT) for 5 min (Table S9).

### The cut-off value of the developed ELISA

Twenty PEDV IgA antibody negative milk samples were measured using the developed ELISA (Table S10). The mean OD_450_ value of these samples was 0.189 and the standard deviation (SD) value was 0.051. Based on the data, the cut-off value of the developed ELISA was 0.342, meaning that the milk samples with the OD_450_ values ≥ 0.342 were considered PEDV IgA antibody positive.

### The sensitivity of the developed ELISA

**Fig. 5:**
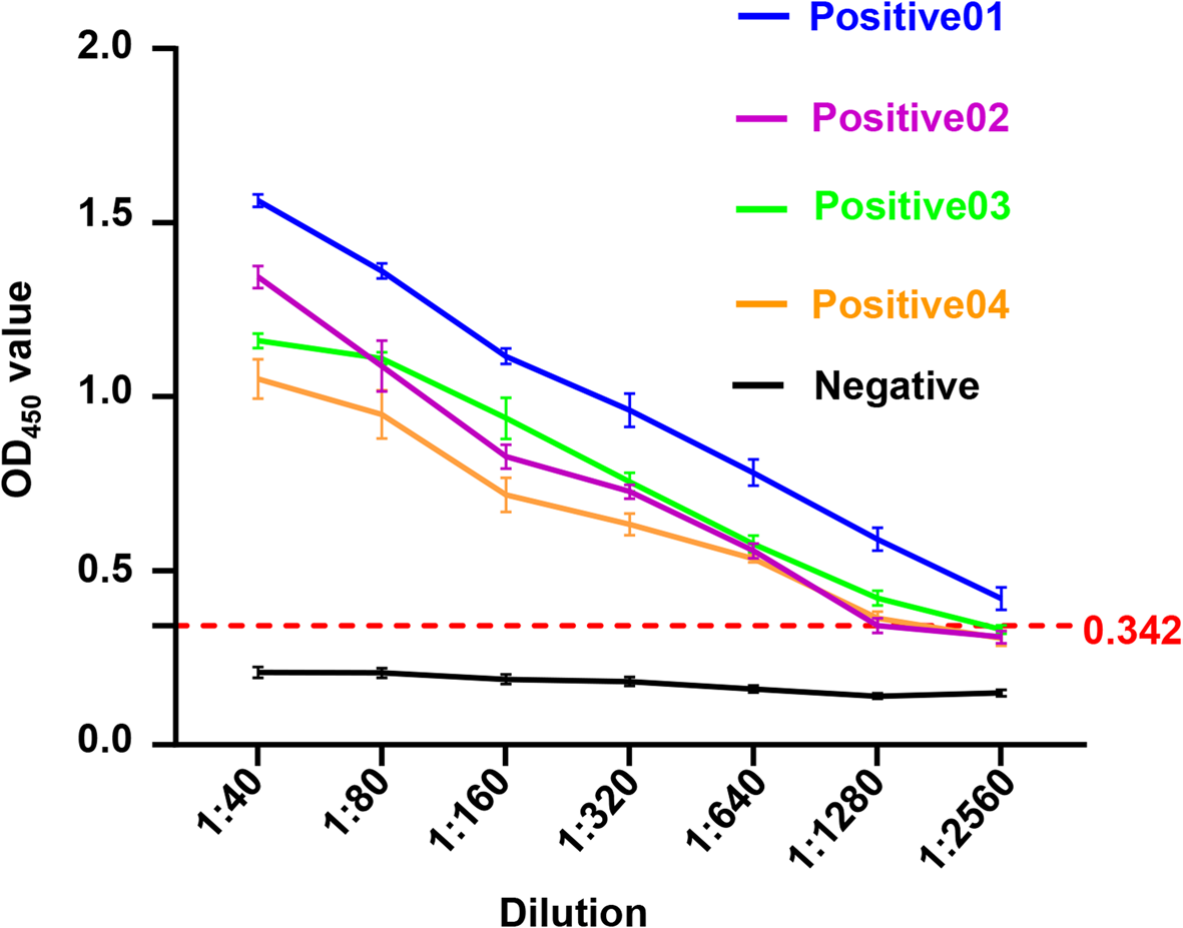
Determination of the sensitivity of the developed ELISA. Four PEDV IgA antibody positive and one negative milk samples were two-fold serially diluted as 1:40, 1:80, 1:160, 1:320, 1:640, 1:1280, 1:2560. The sensitivity of the developed ELISA was determined using the diluted milk samples. The error bars indicate SD values

The sensitivity of the developed ELISA was determined using the diluted milk samples. As shown in Fig. 5 and Table S11, the developed ELISA could detect 1:1280 or even lower diluted PEDV IgA antibody positive milk samples.

### The repeatability of the developed ELISA

The repeatability of the developed ELISA was evaluated with five PEDV IgA antibody positive and one negative milk samples in triplicate on the same plate at the same time and on different plates at different times (Table S12). All the coefficient of variation (CV) values were between 3.60%-8.10%, which were within the allowable range of repeatability (< 10%) [20].

### Clinical milk sample detection by the developed ELISA

In order to determine whether the developed ELISA is applicable to PEDV IgA antibody detection in clinical milk samples, a total of 92 unidentified clinical milk samples were simultaneously tested with the developed ELISA and a commercial ELISA kit (Anheal, Harbin, China). Table 1 showed that the number of the positive samples were 59 detected by the developed ELISA and 56 by the commercial ELISA kit, respectively. The sensitivity of the test was 98.21% (55/56) and the specificity of the test was 88.89% (32/36). Consequently, the developed ELISA highly agreed with the commercial Anheal ELISA kit for detecting PEDV IgA antibodies in clinical milk samples.

**Table 1:** Clinical milk sample detection by the developed ELISA and the commercial Anheal ELISA kit

Clinical milk sample detection	The developed ELISA		
	Positive	Negative	Total
**The commercial Anheal ELISA kit**			
**Positive**	55	1	56
**Negative**	4	32	36
**Total**	59	33	92

## Discussion

PED has emerged or re-emerged as an economically critical enteric disease in pig farming worldwide [21]. The emergence of highly virulent PEDV variants with high morbidity and mortality in neonatal piglets brings about tremendous challenges to prevent and control PED in recent years [9, 22]. Vaccination is still an efficient manner to restrain PED outbreaks. Ideal vaccines for PEDV should be able to induce adequate maternal immunity in immunized sows and passively protect neonatal suckling piglets via colostrum and milk [12].

Among maternal antibodies, IgA antibodies are more resistant to proteolytic digestion in the gastrointestinal tract and have a higher neutralizing ability than IgG and IgM antibodies [23–25]. Current studies have shown that protection of neonatal suckling piglets by maternal antibodies against PEDV infection is mostly attributed to IgA antibodies in milk [13, 26]. As a consequence, PEDV IgA antibody detection in milk is imperative for evaluating vaccine efficacies and neonate immune status.

Although several ELISAs have been established for IgA antibody detection against PEDV in sera [20, 27–29], there are limited ELISAs for IgA antibody detection in colostrum or milk [30–32]. In detail, recombinant or truncated S proteins expressed in eukaryotic/prokaryotic system, nucleocapsid (N) protein expressed in prokaryotic system, or whole PEDV proteins in cell-cultivated virus supernatants were utilized as coating viral antigens in these reports [30–32]. PEDV N protein is inappropriate as coated viral antigen for IgA antibody detection because it shares highly conserved sequences with other Alphacoronaviruses and contains no neutralizing epitopes [33, 34], which may show false results. Whole PEDV proteins coated as viral antigens may also leads to false results due to nonspecific background [32]. In contrast, PEDV S protein is considered as a suitable viral antigen for IgA antibody detection [35]. Consequently, preparation of PEDV S protein with native conformation and high immunoreactivity is of great importance to reduce false results. On the one hand, prokaryotic expression system can’t render proper formation of disulfide bonds, glycosylation or even correct folding to such a membrane protein as PEDV S protein [36]. On the other hand, truncated S protein may not have a comparable immunoreactivity to the entire one [37].

In the current study, we prepared specific mAbs against PEDV S protein, and captured PEDV antigens in inactivated cell-cultivated virus supernatants by the optimal anti-S mAb9. We speculated that the captured PEDV antigens contained the whole virions and S proteins, which were specially and strongly bound by the mAb9 as indicated in Figs. 1, 2, 3 and 4, and therefore probably had a high immunoreactivity. We subsequently developed an ELISA based on captured PEDV antigens by the anti-S mAb. The developed ELISA showed high sensitivity and repeatability in detecting PEDV IgA antibody positive and negative milk samples. The developed ELISA also showed a high coincidence rate with the commercial Anheal ELISA kit for PEDV IgA antibody detection in clinical milk samples. Interestingly, the developed ELISA detected more positive samples than the commercial ELISA kit did. This discrepancy will be validated with other approaches in the future. In fact, we have further evaluated the stability of the developed ELISA and it showed decent detection performance within at least six months (data not shown).

Indeed, more milk samples are required to confirm the clinical performance of the developed ELISA, including the cut-off value and sample to positive ratio. In the meantime, reference IgA antibody positive and negative milk samples are unavailable to us, and as a result, we haven’t conducted comparative assays in the current study. In addition, unlike serum samples, IgA antibody positive milk samples for other swine viruses were difficult to collect, and therefore we haven’t determined the specificity and cross-reactivity of the developed ELISA. However, considering the high specificity and affinity of the anti-S antibody as well as the high immunoreactivity of the captured PEDV antigens, we can anticipate the superiority of the developed ELISA and will addressed this issue in the future. Moreover, the relationships amongst protection efficacy of suckling piglets, neutralizing ability of IgA antibodies in milk and IgA antibody level in milk detected by the developed ELISA should be explored in the future. It’s worth noting that the utilized anti-S mAb was non-neutralizing one (data not shown), and would neither shield the neutralizing epitopes of the captured PEDV antigens nor interfere with neutralization evaluation by IgA antibodies in milk.

## Conclusion

Taken together, an ELISA was developed based on viral antigen capture by a specific anti-S mAb for detecting PEDV IgA antibodies in milk. The developed ELISA showed a high diagnostic sensitivity and repeatability, which provided a potent tool for PEDV surveillance and vaccination evaluation.

## Methods

### Mice, rabbits, cells, and virus

Six to eight-week-old and 16-week-old specific pathogen free female BALB/c mice were purchased from Henan Laboratory Animal Center (Zhengzhou, China). The experimental procedures for mice were authorized and supervised by the Ethical and Animal Welfare Committee of Key Laboratory of Animal Immunology of the Ministry of Agriculture of China (SYXK2021-0003).

Eight-week-old specific pathogen free female New Zealand white rabbits were purchased from Henan Chunying Biotechnology Co., Ltd (Zhengzhou, China). The experimental procedures for rabbits were authorized and supervised by the Ethical and Animal Welfare Committee of Key Laboratory of Animal Immunology of the Ministry of Agriculture of China (LLSC410113).

Vero cell line (CCL-81; kindly provided by Professor Zhanyong Wei of Henan Agricultural University, China, and kept in our laboratory) was cultivated in Dulbecco’s modified Eagle medium (Solarbio, Beijing, China) supplemented with 10% heat-inactivated fetal bovine serum (FBS; Gibco, Grand Island, USA) and antibiotics (100 U/mL penicillin, 100 μg/mL streptomycin; Solarbio) at 37 °C with 5% CO_2_. IPEC-J2 cell line (kindly provided by Professor Zhanyong Wei of Henan Agricultural University, China), SP2/0 myeloma cells (kept in our laboratory) and hybridoma cells (generated in the current study) were maintained in Roswell Park Memorial Institute-1640 medium (Solarbio) with 10% heat-inactivated FBS and antibiotics at 37 °C with 5% CO_2_. The *Drosophila* Schneider 2 (S2) cells stably expressing recombinant PEDV S1 subunit (residues 21–793, the numbering is according to GenBank accession number: KY496315.1) were kept in our laboratory and cultured in Schneider’s insect medium (Gibco) supplemented with 10% heat-inactivated FBS at 28 °C [38].

The prevalent PEDV strain CH/hubei/2016 (GenBank accession number: KY496315.1) was kept in our laboratory and used in the current study [39]. PEDV was propagated in Vero cells according to previous studies [40, 41]. When obvious CPEs were observed with characteristics of cell–cell fusion and syncytium formation at 18 h post infection, the cells were frozen and thawed three times, and then centrifuged at 9600 g at 4 °C for 10 min. The supernatant was filtrated through 0.22 μm filter membranes (Millipore-Merk, Darmstadt, Germany), and stored at -80 °C for further use or inactivated with formaldehyde for viral antigen capture.

### Milk samples

Ten PEDV IgA antibody positive milk samples and 23 PEDV IgA antibody negative milk samples determined by the commercial Anheal ELISA kit (Harbin, China) along with 92 unidentified clinical milk samples were collected from 125 sows by our laboratory. All samples were centrifuged at 6200 g at 4 °C for 5 min. After the lipid layer removed, the middle layer was collected and stored at -20 °C for PEDV IgA antibody detection.

### Preparation of mAbs targeting PEDV

MAbs targeting PEDV were prepared according to our previous study [39]. Briefly, PEDV CH/hubei/2016 virions were purified by tangential flow system and gel filtration chromatography, and utilized as immunogen to immunize six to eight-week-old female BALB/c mice. The spleen cells of the immunized mouse with the highest serum antibody titers were fused with SP2/0 myeloma cells. Supernatants of hybridoma cell cultures were screened by IPMA, and the positive ones were subcloned to obtain the hybridoma cell lines stably secreting anti-PEDV mAbs.

### IPMA screen of anti-PEDV mAbs

PEDV was inoculated in the IPEC-J2 cells with 80% cell confluence on 96-well plates (Corning, Corning, USA). When obvious CPEs appeared, the cells were washed three times with PBS and then fixed with ice-cold methanol containing 3% H_2_O_2_ at -20 °C for 10 min. After fixation, the cells were washed three times with PBS containing 0.05% Tween-20 (PBST) and blocked with 5% skim milk at 37 °C for 1 h. Following washed with PBST, the cells were incubated with the supernatants of hybridoma cell cultures at 37 °C for 30 min. The cells were washed with PBST and added with 1:500 diluted HRP-conjugated goat anti-mouse IgG (Abbkine, Wuhan, China) and stained with 3-amino-9-ethylcarbazole solution (Solarbio) at RT for 5–10 min. The stained cells were examined by light microscopy (Leica, Weztlar, Germany). In parallel, the cells were incubated with PEDV positive and negative sera stored in our laboratory as positive and negative controls, respectively [38, 39].

### Preparation of recombinant PEDV S1 subunit

Recombinant PEDV S1 subunit was prepared as previously described by us [38]. Briefly, recombinant PEDV S1 subunit was expressed by the *Drosophila* S2 cells in Sf-900 II serum-free medium (Invitrogen, Carlsbad, USA), and purified by HisTrap excel prepacked column (GE Healthcare, Fairfield, USA) and HiTrap Q HP prepacked column (GE Healthcare). The purified S1 subunit was applied to 15% SDS-PAGE for analysis.

### Identification of anti-S mAbs by Western blot

The purified recombinant PEDV S1 subunit was subjected to SDS-PAGE and then transferred to polyvinylidene fluoride membranes (PVDFs; Millipore-Merk). The target protein was detected with the supernatants of positive hybridoma cell cultures, HRP-conjugated goat anti-mouse IgG and enhanced chemiluminescence (ECL) plus reagent (NCM Biotech, Suzhou, China). Mock control and recombinant TGEV S protein (kept in our laboratory) were subjected in parallel to assess the specificity of the mAbs.

### IFA analyses of anti-S mAbs

When the confluence reached 80%, Vero cells were inoculated with PEDV and then cultivated at 37 °C until exhibiting obvious CPEs. The cells were fixed with ice-cold methanol and incubated with 3% BSA at 37 °C for 1 h. After washed with PBS, the cells were incubated with the supernatants of positive hybridoma cell cultures and Alexa Fluor 488-donkey anti-mouse IgG (Invitrogen). The cell nuclei were stained using 4’, 6-diamidino-2-phenylindole (DAPI; Solarbio) and then the cells were visualized by fluorescence microscopy (Leica).

### Subtype identification of anti-S mAbs

Subtype identification of anti-S mAbs was conducted according to the manufacturer’s instructions (Proteintech, Wuhan, China).

### Purification of anti-S mAbs

Ascites were prepared via in vivo induction in 16-week-old female BALB/c mice according to conventional procedures. Anti-S mAbs were purified from ascites through (NH_4_)_2_SO_4_ precipitation and Protein G Beads (Thermo Fisher Scientific, Waltham, USA). The purity of anti-S mAbs was analyzed by SDS-PAGE.

### Generation of polyclonal antibodies (pAbs) targeting PEDV

The purified PEDV CH/hubei/2016 virions as described above were utilized as immunogen to immunize eight-week-old New Zealand white rabbits, and preparation of pAbs targeting PEDV was performed according to the procedures in our previous study [42]. A large amount of high-titer pAbs was obtained by heart blood collection method and purified by (NH_4_)_2_SO_4_ precipitation. The generated pAbs were identified using the purified recombinant PEDV S1 subunit by Western blot and further characterized in the IgG class (data not shown).

### Selection of the optimal anti-S mAb for establishing ELISA

The four purified anti-S mAbs were examined to select the optimal one for viral antigen capture. In detail, each purified anti-S mAb (mAb9, mAb10, mAb17, or mAb18) diluted in Solarbio ELISA coating solution (8, 4, 2, 1, 0.5, 0.25, 0.125, and 0.0625 μg/mL; 100 μL/well) was coated on 96-well ELISA plates (NEST, Wuxi, China) at 4 °C overnight. Then, the plates were washed five times with PBST and blocked with 5% skim milk at 37 °C for 2 h. After washed, each coated well was incubated with 100 μL inactivated cell-cultivated PEDV supernatants (1 mg/mL) at 37 °C for 1 h to capture viral antigens. The plates were washed and incubated with the generated rabbit anti-PEDV pAbs at 37 °C for 1 h. Following washed, each well was incubated with 100 μL 1:1000 diluted HRP-conjugated goat anti-rabbit IgG (Abcam, Cambridge, England) as secondary antibody at 37 °C for 30 min. After washed, each well was reacted with 100 μL TMB substrate solution (Solarbio) in dark at RT for 5 min. After added with 100 μL/well of ELISA stop solution (Solarbio), OD_450_ values were immediately measured and recorded on a microplate reader (BMG-Labtech, Offenburg, Germany). The anti-S mAb with the highest OD_450_ values was chosen to establish ELISA. Three replicates of each sample were run, and each experiment was independently repeated three times.

### Optimization of ELISA

The optimal concentrations of the anti-S mAb for coating and the inactivated cell-cultivated PEDV supernatants for viral antigen capture were determined by checkerboard titration [43]. Briefly, the anti-S mAb was two-fold serially diluted as 8, 4, 2, 1, 0.5 and 0.25 μg/mL, and the inactivated cell-cultivated PEDV supernatants were two-fold serially diluted as 4, 2, 1 and 0.5 mg/mL. The concentration was considered optimal when the OD_450_ ratio of PEDV IgA antibody positive to negative milk samples (P/N) was the highest and the OD_450_ value of PEDV IgA antibody positive ones was closest to 1.0. The positive and negative milk samples were diluted as 1:20. The coating condition, blocking solution and condition, dilution of milk samples and HRP-goat anti-pig IgA (Abcam) as secondary antibody, and incubation and reaction time periods were further optimized as described above. Three replicates of each sample were run, and each experiment was independently repeated three times. The mean OD_450_ values presented in Table S2, S3, S4, S5, S6, S7, S8 and S9 were calculated to obtain P/N values according to previous studies [44–46].

### Determination of the cut-off value of the developed ELISA

The cut-off value between PEDV IgA antibody positive and negative milk samples for the developed ELISA was calculated by the mean OD_450_ value plus the three-fold SD value of 20 PEDV IgA antibody negative milk samples [20]. The milk samples with the OD_450_ values ≥ the cut-off value were considered PEDV IgA antibody positive.

### Determination of the sensitivity of the developed ELISA

Four PEDV IgA antibody positive and one negative milk samples were two-fold serially diluted as 1:40, 1:80, 1:160, 1:320, 1:640, 1:1280, 1:2560. The sensitivity of the developed ELISA was determined using the diluted milk samples. Three replicates of each sample were run, and each experiment was independently repeated three times.

### Determination of the repeatability of the developed ELISA

Five PEDV IgA antibody positive and one negative milk samples were applied to evaluate the repeatability of the developed ELISA. Each sample was measured in triplicate on the same plate at the same time and on different plates at different times. CV was calculated as the ratio of the SD value to the mean OD_450_ value [20]. When the CV value was less than 10%, it was considered an acceptable repeatability [20].

### Clinical milk sample detection

A total of 92 unidentified clinical milk samples were simultaneously tested with the developed ELISA and the commercial Anheal ELISA kit to evaluate the detection performance of the developed ELISA. The sensitivity and specificity were calculated according to [the number of true positive samples/(the number of true positive samples + the number of false negative samples)] × 100% and [the number of true negative samples/(the number of true negative samples + the number of false positive samples)] × 100%, respectively [29].

## Supplementary Information

**Additional file 1: Table S1.** Determination of the optimal mAb for ELISA development. **Table S2.** Determination of the optimal concentrations for mAb coating and viral antigen capture. **Table S3.** Determination of the optimal mAb coating condition. **Table S4.** Determination of the optimal blocking solution and condition. **Table S5.** Determination of the optimal dilution of milk samples. **Table S6.** Determination of the optimal incubation time of milk samples. **Table S7.** Determination of the optimal dilution of HRP-conjugated goat anti-pig IgA. **Table S8.** Determination of the optimal incubation time of HRP-conjugated goat anti-pig IgA. **Table S9.** Determination of the optimal reaction condition of TMB substrate solution. **Table S10** Determination of the cut-off value of the developed ELISA. **Table S11.** Determination of the sensitivity of the developed ELISA. **Table S12.** Determination of the repeatability of the developed ELISA.

**Additional file 2.** The original images of Fig. 2A, Fig. 2B and Fig. 4A.

## Data Availability

The data generated and analyzed during the current study are available from the corresponding authors on reasonable request.

## Abbreviations

PED: Porcine epidemic diarrhea
PEDV: PED virus
IgA: Immunoglobulin A
mAb: Monoclonal antibody
S: Spike
ELISA: Enzyme-linked immunosorbent assay
IPMA: Immunoperoxidase monolayer assay
CPE: Cytopathic effect
TGEV: Transmissible gastroenteritis virus
IFA: Indirect immunofluorescence assay
SDS-PAGE: Sodium dodecyl sulfate–polyacrylamide gel electrophoresis
OD_450_: Optical density at 450 nm
PBS: Phosphate buffered saline
BSA: Bovine serum albumin
HRP: Horseradish peroxidase
TMB: 3, 3’, 5, 5’-Tetramethylbenzidine
RT: Room temperature
SD: Standard deviation
CV: Coefficient of variation
N: Nucleocapsid
FBS: Fetal bovine serum
PBST: PBS containing 0.05% Tween-20
PVDF: Polyvinylidene fluoride membranes
ECL: Enhanced chemiluminescence
DAPI: 4’, 6-Diamidino-2-phenylindole
pAbs: Polyclonal antibodies

## References

[1] Jung K, Saif LJ (2015). Porcine epidemic diarrhea virus infection: Etiology, epidemiology, pathogenesis and immunoprophylaxis. Vet J.

[2] Alvarez J, Sarradell J, Morrison R, Perez A (2015). Impact of porcine epidemic diarrhea on performance of growing pigs. PLoS ONE.

[3] Annamalai T, Saif LJ, Lu Z, Jung K (2015). Age-dependent variation in innate immune responses to porcine epidemic diarrhea virus infection in suckling versus weaned pigs. Vet Immunol Immunopathol.

[4] Wood EN (1977). An apparently new syndrome of porcine epidemic diarrhoea. Vet Rec.

[5] Song D, Park B (2012). Porcine epidemic diarrhoea virus: A comprehensive review of molecular epidemiology, diagnosis, and vaccines. Virus Genes.

[6] Wang D, Fang L, Xiao S (2016). Porcine epidemic diarrhea in China. Virus Res.

[7] Sun RQ, Cai RJ, Chen YQ, Liang PS, Chen DK, Song CX (2012). Outbreak of porcine epidemic diarrhea in suckling piglets. China Emerg Infect Dis.

[8] Huang YW, Dickerman AW, Pineyro P, Li L, Fang L, Kiehne R, Opriessnig T, Meng XJ (2013). Origin, evolution, and genotyping of emergent porcine epidemic diarrhea virus strains in the United States. mBio..

[9] Lin CM, Saif LJ, Marthaler D, Wang Q (2016). Evolution, antigenicity and pathogenicity of global porcine epidemic diarrhea virus strains. Virus Res.

[10] Jung K, Saif LJ, Wang Q (2020). Porcine epidemic diarrhea virus (PEDV): An update on etiology, transmission, pathogenesis, and prevention and control. Virus Res.

[11] Li Z, Ma Z, Li Y, Gao S, Xiao S (2020). Porcine epidemic diarrhea virus: Molecular mechanisms of attenuation and vaccines. Microb Pathog.

[12] Langel SN, Paim FC, Lager KM, Vlasova AN, Saif LJ (2016). Lactogenic immunity and vaccines for porcine epidemic diarrhea virus (PEDV): Historical and current concepts. Virus Res.

[13] Song Q, Stone S, Drebes D, Greiner LL, Dvorak CMT, Murtaugh MP (2016). Characterization of anti-porcine epidemic diarrhea virus neutralizing activity in mammary secretions. Virus Res.

[14] Klobasa F, Werhahn E, Butler JE (1987). Composition of sow milk during lactation. J Anim Sci.

[15] Kocherhans R, Bridgen A, Ackermann M, Tobler K (2001). Completion of the porcine epidemic diarrhoea coronavirus (PEDV) genome sequence. Virus Genes.

[16] Bosch BJ, van der Zee R, de Haan CA, Rottier PJ (2003). The coronavirus spike protein is a class I virus fusion protein: Structural and functional characterization of the fusion core complex. J Virol.

[17] Liu C, Tang J, Ma Y, Liang X, Yang Y, Peng G, Qi Q, Jiang S, Li J, Du L (2015). Receptor usage and cell entry of porcine epidemic diarrhea coronavirus. J Virol.

[18] Li C, Li W, Lucio de Esesarte E, Guo H, van den Elzen P, Aarts E, van den Born E, Rottier PJM, Bosch BJ (2017). Cell attachment domains of the porcine epidemic diarrhea virus spike protein are key targets of neutralizing antibodies. J Virol..

[19] Okda FA, Lawson S, Singrey A, Nelson J, Hain KS, Joshi LR, Christopher-Hennings J, Nelson EA, Diel DG (2017). The S2 glycoprotein subunit of porcine epidemic diarrhea virus contains immunodominant neutralizing epitopes. Virology.

[20] Lin H, Zhou H, Gao L, Li B, He K, Fan H (2018). Development and application of an indirect ELISA for the detection of antibodies to porcine epidemic diarrhea virus based on a recombinant spike protein. BMC Vet Res.

[21] Lee C (2015). Porcine epidemic diarrhea virus: An emerging and re-emerging epizootic swine virus. Virol J.

[22] Li W, Li H, Liu Y, Pan Y, Deng F, Song Y, Tang X, He Q (2012). New variants of porcine epidemic diarrhea virus, China, 2011. Emerg Infect Dis.

[23] Brown WR, Newcomb RW, Ishizaka K (1970). Proteolytic degradation of exocrine and serum immunoglobulins. J Clin Invest.

[24] van Egmond M, Damen CA, van Spriel AB, Vidarsson G, van Garderen E, van de Winkel JG (2001). IgA and the IgA Fc receptor. Trends Immunol.

[25] Kaetzel CS (2005). The polymeric immunoglobulin receptor: Bridging innate and adaptive immune responses at mucosal surfaces. Immunol Rev.

[26] Poonsuk K, Giménez-Lirola LG, Zhang J, Arruda P, Chen Q, Correa da Silva Carrion L, Magtoto R, Pineyro P, Sarmento L, Wang C (2016). Does circulating antibody play a role in the protection of piglets against porcine epidemic diarrhea virus?. PLoS One..

[27] Gerber PF, Opriessnig T (2015). Detection of immunoglobulin (Ig) A antibodies against porcine epidemic diarrhea virus (PEDV) in fecal and serum samples. MethodsX.

[28] Shan Y, Gao Q, Mao J, Zheng J, Xu X, Zhang C, Huang X, Xu J, Shi F, Yue M (2022). Establishment of enzyme-linked immunosorbent assays based on recombinant S1 and its truncated proteins for detection of PEDV IgA antibody. BMC Vet Res.

[29] Wang K, Hu Z, Fan M, Shao Z, Yu Q, Li X (2022). Development of an indirect ELISA to detect PEDV specific IgA antibody based on a PEDV epidemic strain. BMC Vet Res.

[30] Gerber PF, Gong Q, Huang YW, Wang C, Holtkamp D, Opriessnig T (2014). Detection of antibodies against porcine epidemic diarrhea virus in serum and colostrum by indirect ELISA. Vet J.

[31] Chang CY, Peng JY, Cheng YH, Chang YC, Wu YT, Tsai PS, Chiou HY, Jeng CR, Chang HW (2019). Development and comparison of enzyme-linked immunosorbent assays based on recombinant trimeric full-length and truncated spike proteins for detecting antibodies against porcine epidemic diarrhea virus. BMC Vet Res.

[32] Srijangwad A, Tripipat T, Saeng-Chuto K, Jermsujarit P, Tantituvanont A, Okabayashi T, Nilubol D (2021). Development and validation of indirect ELISA for antibody detection against different protein antigens of porcine epidemic diarrhea virus in the colostrum and milk of sows. J Immunol Methods.

[33] Hou XL, Yu LY, Liu J (2007). Development and evaluation of enzyme-linked immunosorbent assay based on recombinant nucleocapsid protein for detection of porcine epidemic diarrhea (PEDV) antibodies. Vet Microbiol.

[34] Li Z, Chen F, Yuan Y, Zeng X, Wei Z, Zhu L, Sun B, Xie Q, Cao Y, Xue C (2013). Sequence and phylogenetic analysis of nucleocapsid genes of porcine epidemic diarrhea virus (PEDV) strains in China. Arch Virol.

[35] Lei XM, Yang YL, He YQ, Peng L, Zhao P, Xu SY, Cao H, Fang P, Qiu W, Qin P (2019). Specific recombinant proteins of porcine epidemic diarrhea virus are immunogenic, revealing their potential use as diagnostic markers. Vet Microbiol.

[36] Piao DC, Shin DW, Kim IS, Li HS, Oh SH, Singh B, Maharjan S, Lee YS, Bok JD, Cho CS (2016). Trigger factor assisted soluble expression of recombinant spike protein of porcine epidemic diarrhea virus in Escherichia coli. BMC Biotechnol.

[37] Utiger A, Tobler K, Bridgen A, Suter M, Singh M, Ackermann M (1995). Identification of proteins specified by porcine epidemic diarrhoea virus. Adv Exp Med Biol.

[38] Sun YG, Li R, Jiang L, Qiao S, Zhi Y, Chen XX, Xie S, Wu J, Li X, Deng R (2018). Characterization of the interaction between recombinant porcine aminopeptidase N and spike glycoprotein of porcine epidemic diarrhea virus. Int J Biol Macromol.

[39] Sun YG, Li R, Xie S, Qiao S, Li Q, Chen XX, Deng R, Zhang G (2019). Identification of a novel linear B-cell epitope within the collagenase equivalent domain of porcine epidemic diarrhea virus spike glycoprotein. Virus Res.

[40] Hofmann M, Wyler R (1988). Propagation of the virus of porcine epidemic diarrhea in cell culture. J Clin Microbiol.

[41] Kusanagi K, Kuwahara H, Katoh T, Nunoya T, Ishikawa Y, Samejima T, Tajima M (1992). Isolation and serial propagation of porcine epidemic diarrhea virus in cell cultures and partial characterization of the isolate. J Vet Med Sci.

[42] Geng R, Sun Y, Li R, Yang J, Ma H, Qiao Z, Lu Q, Qiao S, Zhang G (2022). Development of a p72 trimer-based colloidal gold strip for detection of antibodies against African swine fever virus. Appl Microbiol Biotechnol.

[43] Zhang Y, Xu G, Zhang L, Zhao J, Ji P, Li Y, Liu B, Zhang J, Zhao Q, Sun Y (2020). Development of a double monoclonal antibody-based sandwich enzyme-linked immunosorbent assay for detecting canine distemper virus. Appl Microbiol Biotechnol.

[44] Sheng Y, Wang K, Lu Q, Ji P, Liu B, Zhu J, Liu Q, Sun Y, Zhang J, Zhou EM (2019). Nanobody-horseradish peroxidase fusion protein as an ultrasensitive probe to detect antibodies against Newcastle disease virus in the immunoassay. J Nanobiotechnology.

[45] Duan H, Chen X, Zhao J, Zhu J, Zhang G, Fan M, Zhang B, Wang X, Sun Y, Liu B (2021). Development of a nanobody-based competitive enzyme-linked immunosorbent assay for efficiently and specifically detecting antibodies against Genotype 2 porcine reproductive and respiratory syndrome viruses. J Clin Microbiol.

[46] Zhao J, Zhu J, Wang Y, Yang M, Zhang Q, Zhang C, Nan Y, Zhou EM, Sun Y, Zhao Q (2022). A simple nanobody-based competitive ELISA to detect antibodies against African swine fever virus. Virol Sin.

