# Supplementary material for: Development of an enzyme-linked immunosorbent assay based on viral antigen capture by anti-spike glycoprotein monoclonal antibody for detecting immunoglobulin A antibodies against porcine epidemic diarrhea virus in milk
Source: BMC Vet Res. 2023 Feb 11;19:46. doi: 10.1186/s12917-023-03605-4 (PMC9921583; doi:10.1186/s12917-023-03605-4)
Supplement: Supplementary file 1 — Additional file 1: Table S1. Determination of the optimal mAb for ELISA development. Table S2. Determination of the optimal concentrations for mAb coating and viral antigen capture. Table S3. Determination of the optimal mAb coating condition. Table S4. Determination of the optimal blocking solution and condition. Table S5. Determination of the optimal dilution of milk samples. Table S6. Determination of the optimal incubation time of milk samples. Table S7. Determination of the optimal dilution of HRP-conjugated goat anti-pig IgA. Table S8. Determination of the optimal incubation time of HRP-conjugated goat anti-pig IgA. Table S9. Determination of the optimal reaction condition of TMB substrate solution. Table S10 Determination of the cut-off value of the developed ELISA. Table S11. Determination of the sensitivity of the developed ELISA. Table S12. Determination of the repeatability of the developed ELISA. [file 12917_2023_3605_MOESM1_ESM.pdf]

Development of an enzyme-linked immunosorbent assay based on viral antigen capture by anti-spike glycoprotein monoclonal antibody for detecting immunoglobulin A antibodies against porcine epidemic diarrhea virus in milk

Rui Li[1†], Ying Wen[1†], Lei Yang[1], Qi-sheng Qian[1], Xin-xin Chen[1], Jia-qing Zhang[2], Xuewu Li[1], Bao-song Xing[2], Songlin Qiao[1*], Gaiping Zhang[1*]

[1] Key Laboratory of Animal Immunology of the Ministry of Agriculture, Henan Provincial Key Laboratory of Animal Immunology, Henan Academy of Agricultural Sciences, Zhengzhou 450002, Henan, China;

[2] Institute of Animal Husbandry and Veterinary Science, Henan Academy of Agricultural Sciences, Zhengzhou 450002, Henan, China.

[†] Rui Li and Ying Wen contributed equally to this work.

[*] **Correspondence:**

Songlin Qiao: 81615336@qq.com

Gaiping Zhang: zhanggaip@126.com

Table S1 Determination of the optimal mAb for ELISA development

Dilution		8	4	2	1	0.5	0.25	0.125	0.0625
mAb9	Mean OD_{450}	3.222	2.655	2.372	1.858	1.232	1.054	0.813	0.580
	SD	0.071	0.038	0.031	0.036	0.025	0.011	0.016	0.037
mAb10	Mean OD_{450}	2.807	2.481	2.161	1.307	0.779	0.586	0.557	0.491
	SD	0.036	0.039	0.063	0.033	0.017	0.023	0.037	0.038
mAb17	Mean OD_{450}	1.759	1.554	1.177	0.873	0.528	0.439	0.389	0.358
	SD	0.049	0.053	0.070	0.049	0.019	0.030	0.021	0.017
mAb18	Mean OD_{450}	1.815	1.648	1.101	0.970	0.552	0.495	0.278	0.223
	SD	0.033	0.041	0.022	0.048	0.019	0.022	0.019	0.015

Table S2 Determination of the optimal concentrations for mAb coating and viral antigen capture

Milk samples	PEDV antigens (mg/mL)		MAb9 coating concentrations (μg/mL)					
			8	4	2	1	0.5	0.25
Positive	4	Mean OD_{450}	1.900	1.747	1.644	1.622	1.519	1.284
		SD	0.045	0.048	0.022	0.043	0.019	0.054
	2	Mean OD_{450}	1.883	1.722	1.633	1.544	1.516	1.270
		SD	0.038	0.015	0.019	0.024	0.024	0.048
	1	Mean OD_{450}	1.868	1.659	1.608	1.449	1.451	1.246
		SD	0.057	0.032	0.015	0.009	0.026	0.032
	0.5	Mean OD_{450}	1.742	1.607	1.575	1.461	1.417	1.155
		SD	0.046	0.017	0.046	0.038	0.011	0.016
Negative	4	Mean OD_{450}	0.275	0.230	0.208	0.203	0.192	0.188
		SD	0.020	0.011	0.017	0.017	0.011	0.005
	2	Mean OD_{450}	0.263	0.225	0.205	0.193	0.186	0.181
		SD	0.009	0.010	0.012	0.006	0.005	0.010
	1	Mean OD_{450}	0.240	0.216	0.204	0.187	0.173	0.162
		SD	0.022	0.020	0.016	0.005	0.006	0.012
	0.5	Mean OD_{450}	0.227	0.207	0.180	0.171	0.165	0.155
		SD	0.016	0.018	0.014	0.013	0.015	0.012
P/N	4		6.909	7.596	7.904	7.990	7.911	6.830
	2		7.160	7.653	7.966	8.000	8.151	7.017
	1		7.783	7.681	7.882	7.749	8.387	7.691
	0.5		7.674	7.763	8.750	8.544	8.588	7.452

Table S3 Determination of the optimal mAb coating condition

Coating condition		Positive	Negative	P/N
4 °C 12 h	Mean OD_{450}	1.565	0.178	8.792
	SD	0.031	0.001	
4 °C 16 h	Mean OD_{450}	1.722	0.213	8.085
	SD	0.016	0.007	
4 °C 20 h	Mean OD_{450}	1.828	0.277	6.599
	SD	0.029	0.014	

Table S4 Determination of the optimal blocking solution and condition

Blocking solution		Blocking condition										
		37 °C 60 min			37 °C 90 min				37 °C 120 min			
		Positive	Negative	P/N		Positive	Negative	P/N		Positive	Negative	P/N
5% skim milk	Mean OD_{450}	0.851	0.124	6.863	Mean OD_{450}	1.007	0.155	6.497	Mean OD_{450}	1.176	0.169	6.959
	SD	0.014	0.007		SD	0.024	0.008		SD	0.056	0.003	
5% BSA	Mean OD_{450}	0.812	0.117	6.940	Mean OD_{450}	1.049	0.139	7.547	Mean OD_{450}	1.059	0.152	6.967
	SD	0.015	0.005		SD	0.040	0.011		SD	0.009	0.005	

Table S5 Determination of the optimal dilution of milk samples

Dilution		1:20	1:40	1:80	1:160	1:320	1:640	1:1280
Positive	Mean OD_{450}	1.830	1.863	1.335	1.122	1.088	0.678	0.638
	SD	0.028	0.055	0.036	0.059	0.036	0.020	0.050
Negative	Mean OD_{450}	0.183	0.144	0.126	0.153	0.101	0.093	0.089
	SD	0.013	0.006	0.008	0.011	0.008	0.003	0.007
P/N		10.000	12.938	10.596	7.333	10.772	7.290	7.169

Table S6 Determination of the optimal incubation time of milk samples

Incubation time		30 min	60 min	90 min	120 min
Positive	Mean OD_{450}	1.455	1.469	1.589	1.662
	SD	0.070	0.013	0.016	0.032
Negative	Mean OD_{450}	0.146	0.180	0.188	0.201
	SD	0.003	0.002	0.002	0.010
P/N		9.966	8.161	8.452	8.269

Table S7 Determination of the optimal dilution of HRP-conjugated goat anti-pig IgA

Dilution		1:20000	1:40000	1:80000	1:160000
Positive	Mean OD_{450}	1.419	0.781	0.374	0.213
	SD	0.026	0.036	0.025	0.013
Negative	Mean OD_{450}	0.184	0.124	0.082	0.066
	SD	0.011	0.010	0.003	0.008
P/N		7.712	6.298	4.561	3.227

Table S8 Determination of the optimal incubation time of HRP-conjugated goat anti-pig IgA

Incubation time		30 min	45 min	60 min
Positive	Mean OD_{450}	1.295	1.511	1.642
	SD	0.008	0.026	0.007
Negative	Mean OD_{450}	0.163	0.199	0.208
	SD	0.005	0.004	0.007
P/N		7.945	7.593	7.894

Table S9 Determination of the optimal reaction condition of TMB substrate solution

Reaction condition		3 min	5 min	10 min
Positive	Mean OD_{450}	1.058	1.436	1.975
	SD	0.012	0.005	0.012
Negative	Mean OD_{450}	0.123	0.158	0.234
	SD	0.003	0.002	0.004
P/N		8.602	9.089	8.440

Table S10 Determination of the cut-off value of the developed ELISA

Negative	OD_{450} value									
01-10	0.151	0.142	0.161	0.155	0.159	0.149	0.130	0.201	0.324	0.178
11-20	0.264	0.199	0.171	0.176	0.170	0.267	0.184	0.198	0.148	0.255
Mean OD_{450}	0.189									
SD	0.051									

Table S11 Determination of the sensitivity of the developed ELISA

Dilution		1:40	1:80	1:160	1:320	1:640	1:1280	1:2560
Positive01	Mean OD_{450}	1.563	1.361	1.116	0.961	0.782	0.590	0.420
	SD	0.018	0.022	0.022	0.048	0.038	0.033	0.032
Positive02	Mean OD_{450}	1.344	1.088	0.828	0.727	0.557	0.342	0.309
	SD	0.032	0.073	0.034	0.02	0.021	0.021	0.017
Positive03	Mean OD_{450}	1.161	1.110	0.938	0.755	0.577	0.421	0.331
	SD	0.021	0.017	0.059	0.027	0.023	0.021	0.012
Positive04	Mean OD_{450}	1.051	0.949	0.718	0.633	0.535	0.363	0.306
	SD	0.056	0.069	0.049	0.031	0.011	0.020	0.022
Negative	Mean OD_{450}	0.208	0.206	0.188	0.182	0.160	0.140	0.149
	SD	0.016	0.014	0.013	0.012	0.010	0.008	0.009

Table S12 Determination of the repeatability of the developed ELISA

	Positive01	Positive02	Positive03	Positive04	Positive05	Negative
1	0.762	1.029	1.270	1.481	1.245	0.180
2	0.718	1.132	1.135	1.409	1.176	0.173
3	0.752	1.229	1.227	1.465	1.119	0.162
4	0.779	1.197	1.078	1.423	1.072	0.168
5	0.791	1.035	1.213	1.335	1.059	0.173
6	0.714	1.030	1.060	1.442	1.065	0.179
Mean OD_{450}	0.753	1.109	1.164	1.426	1.123	0.173
SD	0.031	0.090	0.086	0.052	0.075	0.007
CV	4.10%	8.10%	7.40%	3.60%	6.68%	4.00%
