# Supplementary material for: Development of an enzyme-linked immunosorbent assay based on viral antigen capture by anti-spike glycoprotein monoclonal antibody for detecting immunoglobulin A antibodies against porcine epidemic diarrhea virus in milk
Source: BMC Vet Res. 2023 Feb 11;19:46. doi: 10.1186/s12917-023-03605-4 (PMC9921583; doi:10.1186/s12917-023-03605-4)

Development of an enzyme-linked immunosorbent assay based on viral antigen capture by anti-spike glycoprotein monoclonal antibody for detecting immunoglobulin A antibodies against porcine epidemic diarrhea virus in milk

Rui Li[1†], Ying Wen[1†], Lei Yang[1], Qi-sheng Qian[1], Xin-xin Chen[1], Jia-qing Zhang[2], Xuewu Li[1], Bao-song Xing[2], Songlin Qiao[1*], Gaiping Zhang[1*]

[1] Key Laboratory of Animal Immunology of the Ministry of Agriculture, Henan Provincial Key Laboratory of Animal Immunology, Henan Academy of Agricultural Sciences, Zhengzhou 450002, Henan, China;

[2] Institute of Animal Husbandry and Veterinary Science, Henan Academy of Agricultural Sciences, Zhengzhou 450002, Henan, China.

[†] Rui Li and Ying Wen contributed equally to this work.

[*] Correspondence:

Songlin Qiao: 81615336@qq.com

Gaiping Zhang: zhanggaip@126.com

The original image of Fig. 2A

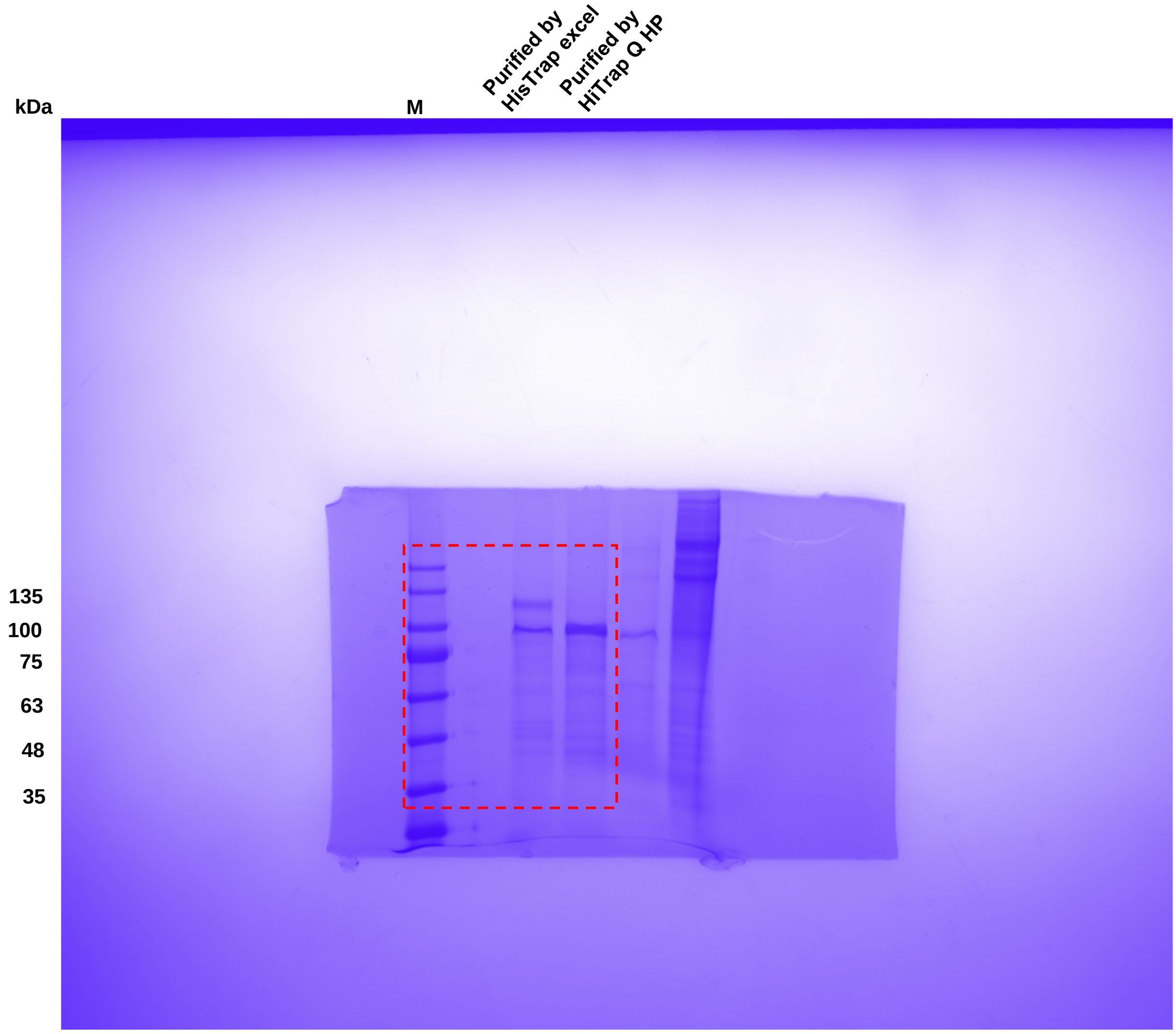

The original image of Fig. 2B

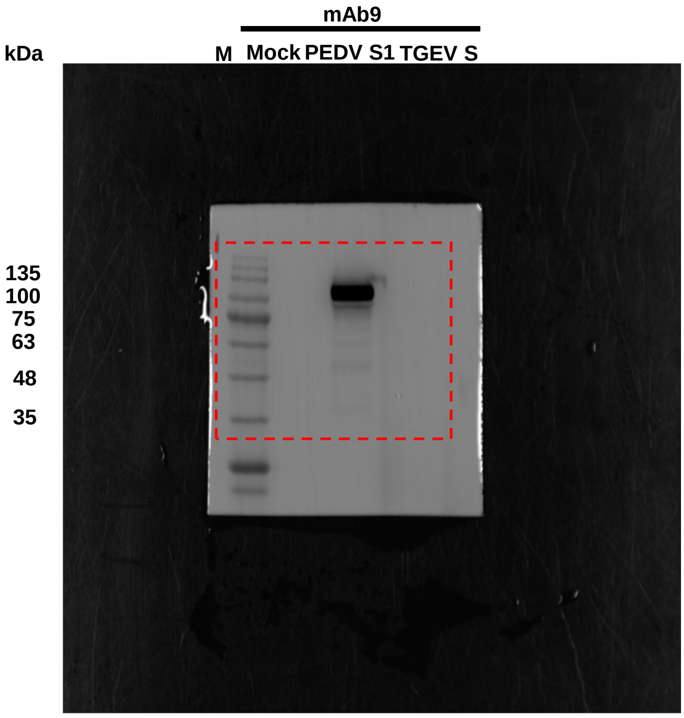

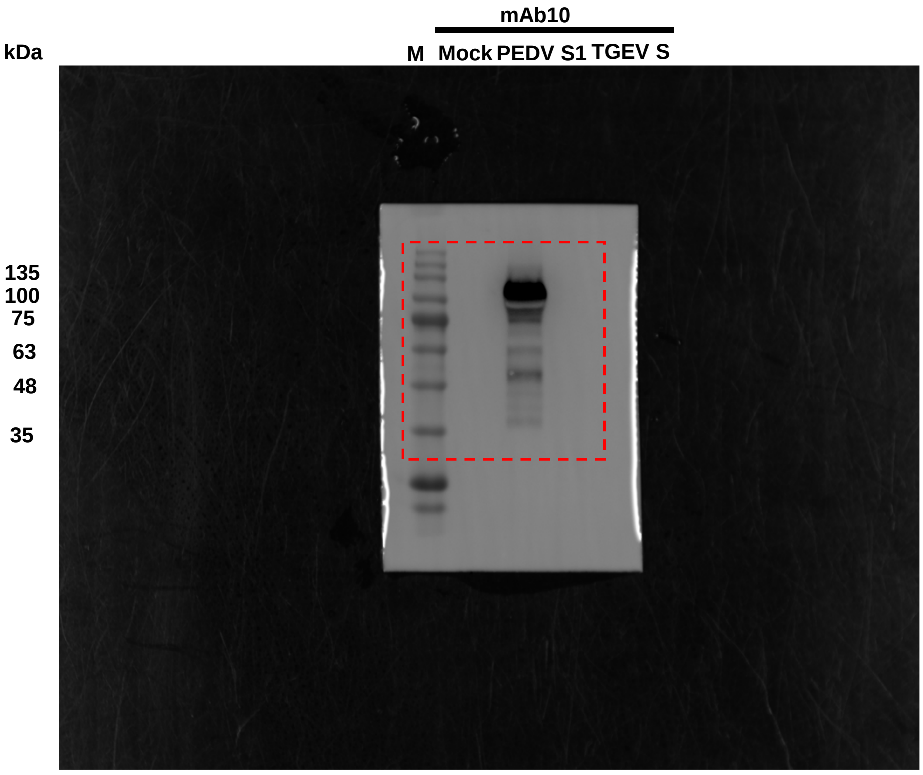

The original image of Fig. 2B

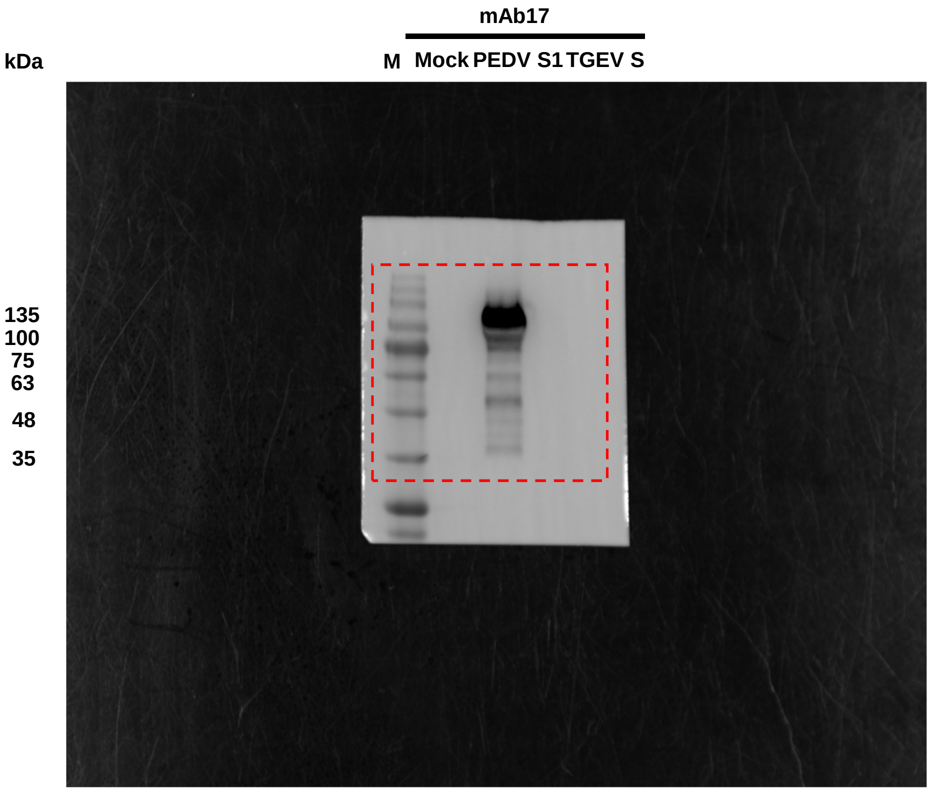

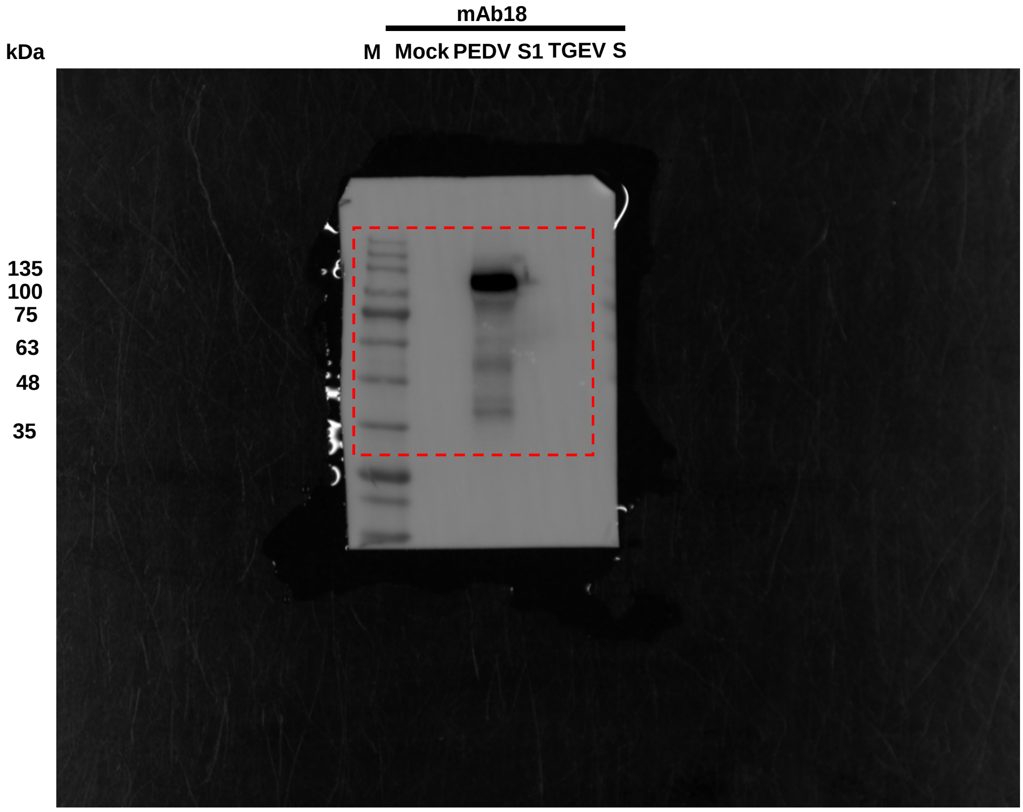

The original image of Fig. 4A

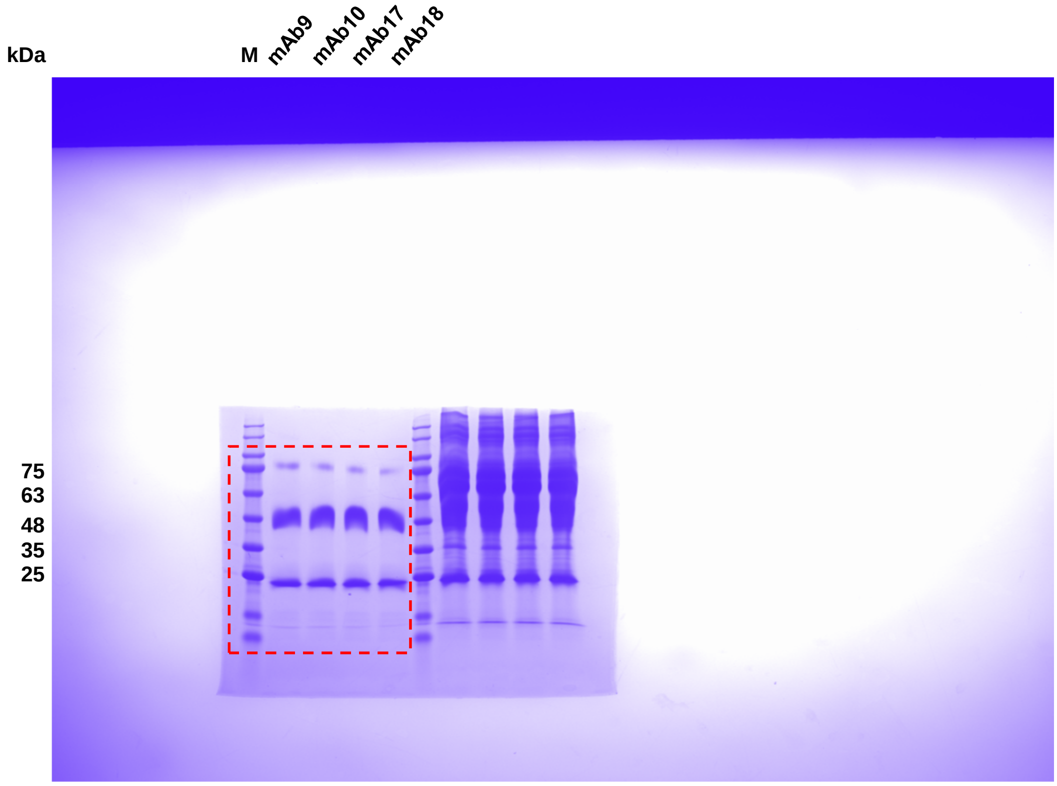

Supplement: Supplementary file 2 — Additional file 2. The original images of Fig. 2A, Fig. 2B and Fig. 4A. [file 12917_2023_3605_MOESM2_ESM.pdf]
